# Inhibition of cell growth by cellular differentiation into adipocyte-like cells in dexamethasone sensitive cancer cell lines

**DOI:** 10.1080/19768354.2018.1476408

**Published:** 2018-06-18

**Authors:** Hea-In Kim, Sun-Ha Moon, Won-Cheol Lee, Hyeon-Jeong Lee, Sharath Belame Shivakumar, Sung-Ho Lee, Bong-Wook Park, Gyu-Jin Rho, Byeong-Gyun Jeon

**Affiliations:** a Department of Biology Education, Gyeongsang National University, Jinju, Republic of Korea; b OBS/Theriogenology and Biotechnology, Gyeongsang National University, Jinju, Republic of Korea; c Division of Life Science, Gyeongsang National University, Jinju, Republic of Korea; d Department of Oral and Maxillofacial Surgery, Changwon Gyeongsang National University Hospital, Changwon, Republic of Korea; e Research Institute of Education, Gyeongsang National University, Jinju, Republic of Korea

**Keywords:** Human, cancer, dexamethasone, cell differentiation, adipocytes

## Abstract

The stress responses in human body lead to secretion of cortisol hormone. The present study investigated the cellular responses on cell growth and cellular differentiation into adipocytes by exposure of synthetic stress hormone, dexamethasone (DEX) in various human cancer and normal cells. After prolonged exposure of cells with 1 μg/ml DEX for 2 weeks, population doubling time (PDT) was significantly (*P* < .05) increased by inhibited cell growth in A-549 and MCF-7 cancer cells, and was unchanged in MDA-MB-231 cancer cells, normal MRC-5 fibroblasts, umbilical cord matrix-derived mesenchymal stem cells (UCMSCs) and dental papilla tissue-derived mesenchymal stem cells (DSCs). Whereas, PDT was significantly (*P* < .05) decreased in U87-MG cancer cells by increased cell growth. Glucose uptake was significantly (*P* < .05) increased in all the cancer cell lines compared to that in normal cell lines. Further, adiposome-like vesicles were noted in A-549 and MCF-7 cancer cells indicating retarded cell growth by DEX treatment, and the vesicles were stained with Oil-Red O solution. Further, the expression of adipocyte-specific genes such as glucose transporter type 4 (GLUT4), glucocorticoid receptors *β* (GR*β*) and peroxisome proliferator-activated receptor *γ* (PPAR*γ*) were significantly (*P* < .05) increased in A-549 and MCF-7 with lipid vesicles. The level of telomerase activity was found to be significantly (*P* < .05) downregulated in DEX-treated A-549 and MCF-7 cancer cells. Our results have clearly shown that DEX treatment induces inhibition of cell growth by differentiating into adipocyte-like cells in dexamethasone sensitive cancer cells.

## Introduction

The secretion of adrenocorticotropic hormone by the anterior pituitary gland is stimulated under stress conditions, and this further increase the secretion of glucocorticoids (GCs) often referred to as corticosteroids from the adrenal gland of almost every vertebrate, including human. The main function of secreted GCs in response to the stress condition is to increase the concentration of blood glucose by stimulating gluconeogenesis resulting in the synthesis of glucose from non-glucose carbohydrate, and through the breakdown of amino acids and lipids (Munck et al. 1984). Otherwise, GCs combined with their receptor (GR) play a major role in the anti-inflammatory response of cells through transcriptional regulation (Busillo and Cidlowski 2013). Previously, it has been demonstrated that lymphocyte proliferation, such as T-cells, is inhibited by decreased production of T cell growth factor which play a role in the production and development of the T-cells (Arya et al. 1984). Thus, the GC compounds are conventionally used for the treatment of inflammatory and autoimmune related-diseases and/or the prevention of tissue rejection after transplantation for over half a century (Scheinman et al. 1995; Flammer and Rogatsky 2011). Nevertheless, GCs induces the decreased secretion of several types of interferons and interleukins which are responsible for the interactions and communications between immune cells (Kunicka et al. 1993). Therefore, it has been suggested that exposure to GCs for prolonged time seems to be one of factors inducing cancers via malfunction of immune system induced with stress (Godbout and Glaser 2006). Meanwhile, the inhibition of cell growth and induction of apoptotic cell death are observed in several type of cancer cells after being exposed to GCs (Bailly-Maitre et al. 2001; Qian et al. 2011; Buxant et al. 2015; Jackson et al. 2016). Therefore, GCs are often considered a potential alternative chemotherapeutic drug for cancer treatment and reducing cancer pains in solid tumors and acute lymphoid leukemia by their cytotoxic effects, such as cellular apoptosis (Yennurajalingam et al. 2013; Jackson et al. 2016).

There are several criteria for defining characteristics of cancer cells. Most of the cancer cells exhibit immortalization by undergoing limitless cell divisions even in the absence of growth signals, resistance to apoptotic cell death, loss of contact inhibition and invasion into other tissues (Hanahan and Weinberg 2000). Furthermore, it has been known that the malignant tumor or cancer cells are relatively undifferentiated cells having capacity to differentiate into several somatic cell types when compare to benign tumor cells, and the differentiation of these cancer cells into specific type of cells is also related to the loss of tumorous characteristics (Reya et al. 2001). It is a well-known fact that increased glucose uptake and glycolysis is exhibited in most of the cancer cells (Shaw 2006). The exposure of cancer cells to GCs which play a major role in glucose metabolism may induce the alternation of cellular glucose metabolism. In the previous reports, GCs are most widely used in the cellular differentiation of mesenchymal stem cells into adipocytes (Jeon et al. 2011b, 2015). Thus, treatment of cancer cells with GCs at a specific concentration may force them to differentiate into adipocytes while losing tumorous characteristics. The treatment of cancer cell lines with GCs displayed cytotoxic effects including inhibition of cell proliferation and cellular apoptosis in previous reports, however, the effects on cells pertaining to adipogenesis is unclear and yet to be investigated. Cancer cells exhibit unlimited cell proliferation capacity by maintaining telomeric repeats at the DNA ends of linear chromosomes of eukaryotes, and these telomeric repeats are stabilized by highly up-regulated telomerase activity without shortening of these telomeric repeats compared to normal or fully differentiated cell lines having down-regulated telomerase activity at a basal level (Cong et al. 2002; Arnoult and Karlseder 2015). The high level of telomerase activity is also one of the main criteria to define tumorous cells, and anti-tumor drugs for reducing telomerase activity and telomeric repeats have been continually developed and tried for cancer treatment (Hanahan and Weinberg 2000; Jeon et al. 2011a; Arnoult and Karlseder 2015; Kim et al. 2017). However, the cytotoxic effect on telomerase activity is not fully investigated in both cancer and normal cells which are directly treated with GCs of high level until now.

In the present study, different types of cancer and normal cells, including A-549 human lung adenocarcinoma, MDA-MB-231 human breast adenocarcinoma, U87-MG human brain glioblastoma astrocytoma cells, MCF-7 human breast adenocarcinoma, mesenchymal stem cells derived from human umbilical cord matrix (UCMSCs), mesenchymal stem cells derived from human dental papilla tissues (DPSCs) and MRC-5 human fetal fibroblasts were exposed at 1 μg/ml of dexamethasone (DEX), one of the most widely used synthetic glucocorticoids for up to 2 weeks. Mouse 3T3-L1 pre-adipocytes were readily undergone adipogenesis by DEX exposure, and used as an internal control cells for adipogenesis. And then we investigated the effect of DEX treatment on the cell growth by population doubling time, glucose uptake, and cellular differentiation into adipocytes. Further, the level of telomerase activity was assessed by change in the expression level of human telomerase reverse transcriptase (TERT). The senescence-associated *β*-glucosidase (SA *β*-gal) was also assessed in cancer cells treated with 1 μg/ml DEX.

## Materials and methods

### Culture and treatment of cells

The basic medium for cell culture was advanced-Dulbecco’s modified eagle medium (A-DMEM) supplemented with 3% fetal bovine serum and 1.0% penicillin–streptomycin (10,000 IU and 10,000 μg/ml, respectively). The pH and osmolality of the media was 7.4 and 280 mOsm/kg, respectively. Media and other cell culture materials were purchased from Invitrogen (USA) and Sigma (USA), unless otherwise specified. The cancer and normal cell lines used in the present study, including MDA-MB-231 human breast adenocarcinoma, A-549 human lung adenocarcinoma, MCF-7 human breast adenocarcinoma, U87-MG human brain glioblastoma astrocytoma, mouse- derived 3T3-L1 preadipocytes and MRC-5 human fetal fibroblasts were purchased from American Type Culture Collection (ATCC, USA). The human mesenchymal stem cell lines were isolated from umbilical cord matrix tissues (UCMSCs) of newborns, also known as Wharton’s jelly mesenchymal stem cells, and dental papilla tissues (DPSCs) of the extracted third molar tooth of healthy donor, respectively, as previously described (Jeon et al. 2011b, 2015). The stemness characterizations of the isolated USMSCs and DPSCs were fully demonstrated in our previous study (Jeon et al. 2011b, 2015). All cell types were cultured in the basic medium at 37.5°C under 5% CO_2_, and continually sub-cultured at 90% confluent status by changing media twice a week. The dexamethasone (DEX) was dissolved in methanol at 1 mg/ml and the working stock solutions were freshly prepared by diluting with basic culture medium before use. All cell types were exposed to 1 μg/ml of DEX for up to 2 weeks (14 days), and cells were dissociated with 0.25% trypsin and either immediately analyzed or frozen at −80°C for future analysis.

### Analysis of population doubling time (PDT)

The effect of DEX on the inhibition of cell growth was analyzed by the evaluation of PDT. Each cancer and normal cells were seeded at 0.5 × 10^3^ cells/well into a 6-well plate and cultured in A-DMEM media supplemented with 0 μg/ml (untreated control) and 1 μg/ml DEX for 2 weeks at 37.5°C in a humidified atmosphere of 5% CO_2_ in air, and the culture media were regularly changed twice a week. Cells were then collected after trypsinization with 0.25% trypsin and the number of cells was counted using a hemocytometer. The PDT was calculated using the formula PDT = *t* (log 2)/(log *N*_t_–log *N*_o_), where *t* is cell culture duration (hrs), *N*_o_ and *N*_t_ are the cell numbers before and after seeding, respectively.

### Analysis of glucose uptake

The glucose uptake assay was determined by measuring glucose concentration of the medium. Each cancer and normal cells were seeded at 5 × 10^4^ cells into a 25 T culture flask and cultured in A-DMEM media supplemented with 0 μg/ml (untreated control) and 1 μg/ml DEX for 1 week without changing the culture media. After being collected, the glucose concentration of the medium was measured with Accu-Chek blood glucometer and test strip (Roche, Germany) using glucose dehydrogenase assay in ten replicates. The number of cells in each treatment was counted using a hemocytometer. The glucose uptake is represented as consumed glucose concentration (ng/dl) per 1,000 cells.

### Analysis of cellular differentiation into adipocytes

The cells with lipid-like droplets were frequently observed after treatment of 1 μg/ml DEX. To investigate the cellular differentiation into adipocytes, the cells were washed in D-PBS and fixed with 3.7% paraformaldehyde for overnight. Then, the cells were again washed twice with D-PBS and treated with 0.5% Oil Red O solution for staining of adiposomes with neutral triglycerides and lipids for 2 h at room temperature. The frequency of the cells with lipid droplets stained with red color was examined under an inverted microscope (Nikon, Japan).

### Analysis of transcripts by reverse transcription polymerase chain reaction (RT–PCR)

The RT–PCR assay was employed to analyze the expression level of adipogenesis and telomerase-related transcripts. The total RNA from untreated control and DEX-treated cells was purified using RNeasy Micro kit (Qiagen, Germany) as per the protocol provided and quantified using a spectrophotometer (Mecasys, Korea). The cDNA synthesis of the extracted total RNA was performed using Omniscript reverse transcription kit (Qiagen), containing 1 μg total RNA, 2 μl of 10 μM random hexamer, 1 μl of 10 U/μl RNase inhibitor, 2 μl dNTP, 4 U reverse transcriptase in a 20 μl reaction mixture at 42°C for 1 h. Each samples were converted to cDNA in at least three reactions. The expression level of selected transcripts was analyzed by PCR assay and subsequent product intensity on agarose gel. The PCR amplification from cDNA samples was performed in thermal cycler (TaKaRa, Japan) using Maxime-PCR PreMix Kit (iNtRON Biotechnology, Korea) in 30 PCR cycles with each cycle consisting of initial denaturation step at 94°C for 1 min, annealing step at 56–60°C for 30 sec and elongation step at 72°C for 1 min. The PCR reactions contained 2 μl of cDNA sample and 1 μl each of the forward and reverse primer (10 μM), the final volume was adjusted to 20 μl with DEPC water. After PCR amplification, the product size and intensity of the PCR was confirmed on 1% agarose gel using image-processing software (ATTO, Japan). PCR amplification was carried out in triplicates for each cDNA sample. The expression level of the transcripts in each sample was calculated in relative to the expression level of a reference gene glyceraldehyde 3-phosphate dehydrogenase (GAPDH). The sequences of primer used in this study were GAPDH and telomerase reverse transcriptase (TERT) related to telomerase activity were previously described (Kim et al., 2017). The primers for adipogenesis were glucose transporter 4 (GLUT4, sense: ATGCTGCTGCCTCCTATGAA, antisense: CAGTTGGTTGAGCGTCCC), glucocorticoid receptor β (GRβ, sense: GAAGGAAACTCCAGCCAGAAC, antisense: TGAGCGCCAAGATTGTTGG) and peroxisome proliferator-activated receptor γ (PPARγ, sense: CCTATTGACCCAGAAAGCGATT, antisense: CATTACGGAGAGATCCACGGA), and the size of PCR products was 146, 140 and 135 bp, respectively.

### Analysis of telomerase activity by relative-quantitative telomerase repeat amplification protocol (RQ-TRAP)

For the quantification of telomerase activity, the traditional TRAP assay protocol based on PCR and gel electrophoresis was used with minor modification using real time Rotor Gene Q (Qiagen, USA) as previously described by Jeon et al. (2011b). Briefly, the cells in each treatment were collected at 1 × 10^5^ cells per sample and protein was extracted with 400 µl of TRAPeze® 1X CHAPS cell lysis buffer (Millipore, USA) for 30 min on ice. After being centrifuged for 20 min at 12,000 × g at 4°C, 60–70% (by volume) of the supernatant to remove cell debris and DNA was carefully collected to a fresh sample tube and the concentration of total protein in each sample was subsequently measured with a spectrophotometer (Mecasys, Korea). The reaction mixture for RQ-TRAP amplification was prepared with 1 μg total protein of each of the lysed sample, Rotor-Gene^TM^ 2 × SYBR green kit (Qiagen, USA), 0.02 µg of telomerase TS primer and 0.04 µg of anchored return ACX primer in the 20 μl of final volume, and TS and ACX primer were previously described (Kim et al. 2017). Before RQ-TRAP amplification, the reactions were incubated at 30°C for 30 min and subsequently at 94°C for 10 min for denaturation. The RQ-TRAP amplification consisted of 94°C for 30 sec, 60°C for 90 sec and 72°C for 0 sec for 40 cycles. The relative level of telomerase activity in cancer cell lines was calculated against a level of telomerase activity of untreated control MRC-5 fibroblasts with the second derivative method by difference of crossing point (Cp) using Rotor-Gene Q Series Software (Qiagen, USA) in five replicates.

### Analysis of senescence-associated β-galactosidase (SA *β*-gal) activity

The cellular senescence was investigated in the untreated control and 1 μg/ml DEX-treated cells using SA β-gal staining kit (Cell Signaling Technology, USA) as per the manufacturer’s protocol. Briefly, after seeding cancer cells at 0.5 × 10^3^ cells/well into a 6-well plate, the cells were exposed to A-DMEM media supplemented with 0 μg/ml (untreated control) and 1 μg/ml DEX with regular media change twice a week. After culture for up to 2 weeks, the cells were washed with D-PBS, fixed with 1 ml fixative solution for 10–15 min at room temperature, and incubated with SA β-gal substrate solution at 37°C for overnight. And the frequency and intensity of cell with activity of SA β-gal was assessed under an inverted microscope (Nikon, Japan) equipped with CCD camera and image program. The proportion of cells exhibiting a blue color was estimated as positive cell for cellular senescence.

### Statistical analysis

Differences among the cell groups were analyzed by using one-way analysis of variance (ANOVA, SPSS 15.0 version, USA). All data were expressed as mean ± standard error of the mean (SEM). The significant differences were considered when *P* < .05.

## Results

### Analysis of population doubling time

**Figure 1.**
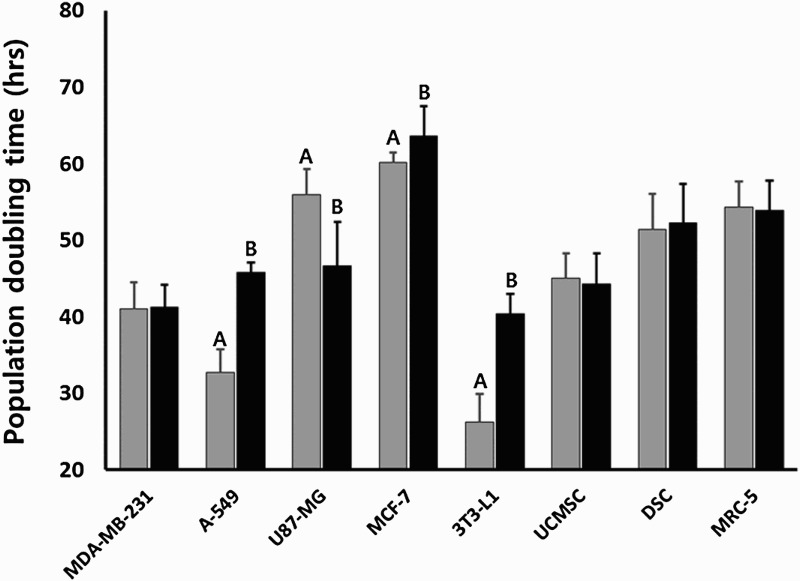
Analysis of PDT in human cancer and normal cell lines of various types exposed to 0 (untreated control, ▪) and 1 μg/ml DEX (▪) for 2 weeks. A and B indicate significant (*P* < .05) difference between untreated control and DEX treatment, respectively.

The cancer cells were exposed to 1 μg/ml DEX for 2 weeks. After counting cell numbers, the population doubling time (PDT) was assessed and the results are shown in Figure 1. The PDT (mean ± SEM) was 41.0 ± 3.45, 32.4 ± 2.98, 56.0 ± 3.35, 60.1 ± 1.29, 45.0 ± 3.28, 51.4 ± 4.68 and 54.3 ± 3.33 h in untreated control MDA-MB-231, A-549, U87-MG, MCF-7, UCMSC, DSC and MRC-5 cell lines in three replicates, respectively. The A-549 cancer cells displayed higher proliferation rate compare to other cell lines. Whereas, the PDT (mean ± SEM) was 41.3 ± 2.87, 45.8 ± 1.26, 46.7 ± 5.76, 63.7 ± 3.89, 44.3 ± 4.01, 52.3 ± 5.02 and 53.9 ± 3.88 h in MDA-MB-231, A-549, U87-MG, MCF-7, UCMSC, DSC and MRC-5 cell lines after treating with DEX, respectively. After prolongedly exposure with 1 μg/ml DEX for 2 weeks, the population doubling time (PDT) was significantly (*P* < .05) increased in A-549 and MCF-7 cancer cells, and PDT was not changed in MDA-MB-231 cancer cells, MRC-5 fibroblasts, UCMSCs and DSCs. Especially, the PDT was highly increased by inhibition of cell growth in mouse 3T3-L1 preadipocytes used as an internal control cells for adipogenesis. Whereas, the PDT was significantly (*P* < .05) decreased in only U87-MG cancer cells by increased cell growth.

### Analysis of glucose uptake

**Figure 2.**
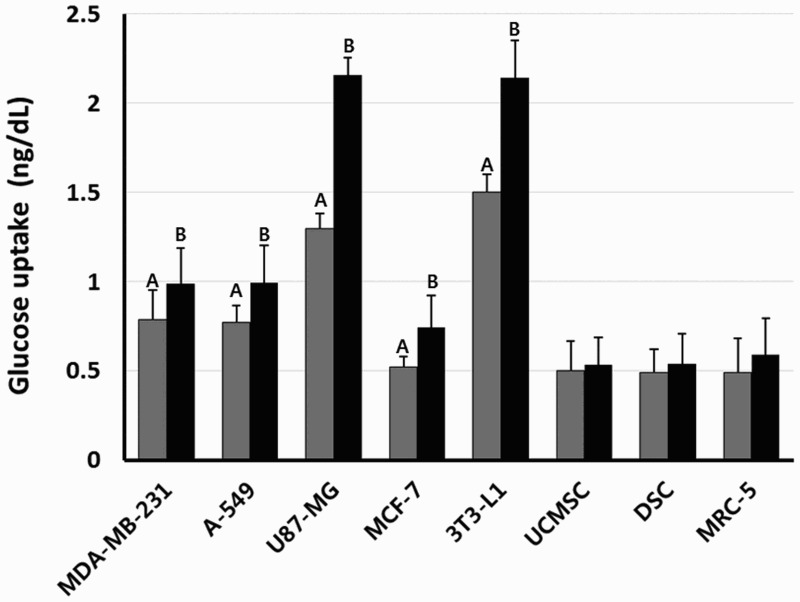
Effect of DEX treatment on glucose uptake in human cancer and normal cell lines of various types. Each cancer and normal cell lines were exposed to A-DMEM 0 (untreated control, ▪) and 1 μg/ml DEX (▪) for 1 week without media change, respectively. Values indicates mean glucose concentration (ng/dl) per 1000 cells consumed during in vitro culture. A and B indicate significant (*P* < .05) difference between untreated control and DEX treatment, respectively.

After DEX treatment, the consumed glucose concentration was measured in each cell lines, as shown in Figure 2. The amount of consumed glucose per 1000 cells was 0.79 ± 0.097, 0.77 ± 0.165, 1.30 ± 0.083, 0.52 ± 0.057, 0.50.0 ± 0.169, 0.49 ± 0.132 and 0.49 ± 0.189 ng/dl in the untreated control MDA-MB-231, A-549, U87-MG, MCF-7, UCMSC, DSC and MRC-5 cell lines, respectively. However, the amount of consumed glucose per 1000 cells was 0.98 ± 0.207, 0.99 ± 0.199, 2.16 ± 0.099, 0.75 ± 0.175, 0.53.0 ± 0.152, 0.54 ± 0.169 and 0.59 ± 0.200 ng/dl in the DEX-treated MDA-MB-231, A-549, U87-MG, MCF-7, UCMSC, DSC and MRC-5 cell lines, respectively. The consumption ratio of glucose was significantly (*P* < .05) increased by DEX treatment in most of the cancer cells lines than those of normal cell lines.

### Analysis of cellular differentiation into adipocytes

**Figure 3.**
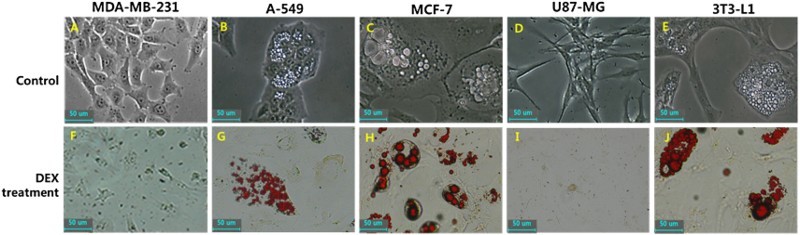
In vitro differentiation into adipocyte-like cells by DEX treatment in human in A-549, MDA-MB-231, MCF-7 and U87-MG cancer cell lines. Mouse 3T3-L1 pre-adipocytes were used as an adipogenic control cells. Morphological alternation was evaluated after DEX treatment for 2 weeks under light microscope (A, B, C, D and E) and adiposomes like-cellular organelles were observed in A-549, MCF-7 cancer cells and 3T3-L1 mouse pre-adipocytes (B, C and E). Further, accumulation of intracellular lipid droplets was stained with oil red O solution (F, G, H, I and J) and adiposomes stained with red spots were also observed in A-549, MCF-7 cancer cells and mouse 3T3-L1 pre-adipocytes (J, H and J). Scale: 50 μm.

**Figure 4.**
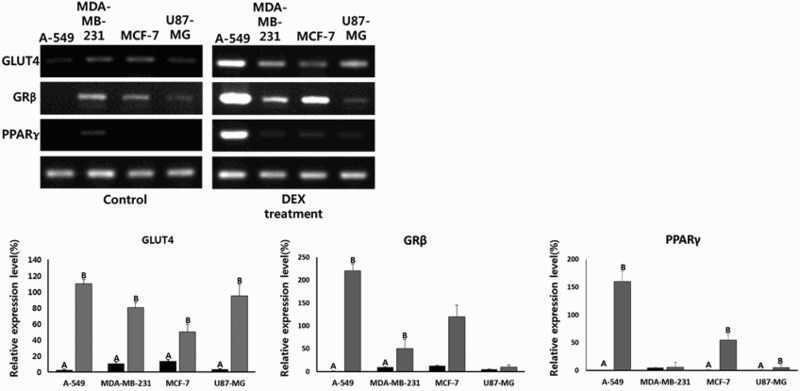
Expression level of GLUT4, GRβ and PPARγ related with adipogenesis by RT-PCR in A-549, MDA-MB-231, MCF-7 and U87-MG cancer cell lines. A and B indicates significant (P<.05) difference between untreated control (▪) and 1 μg/ml DEX treatment (▪), respectively.

Following DEX treatment for up to 2 weeks, the glucose uptake was significantly increased in cancer cells, and the adiposome-like vesicles with fat molecules were frequently observed in A-549 and MCF-7 cancer cells. The adiposome-like vesicles were highly reacted to Oil Red O solution that commonly used for staining of neutral triglycerides and lipids (Figure 3). The A-549 and MCF-7 cancer cells were observed to be readily differentiated into adipocyte-like cells after prolonged exposure of DEX. Further, the expression level of transcripts related to adipogenesis, including GLUT4, GRβ and PPARγ was quantified by RT-PCR as shown in Figure 4. Following DEX treatment, the GLUT4 transcript was significantly (*P* < .05) increased in A-549, MDA-MB-231, MCF-7 and U87-MG cancer cells. The GRβ transcript was significantly (*P* < .05) increased in A-549, MDA-MB-231 and MCF-7 cancer cells. The PPARγ transcript was also significantly (*P* < .05) increased in A-549, MCF-7 and U87-MG cancer cells. However, even though PPARγ transcript in the DEX-treated U87-MG cancer cells was significantly (*P* < .05) increased than that of untreated control counterpart, the expression level of transcript was observed to be detected at an extremely weak level, compared to that in A-549 and MCF-7 cancer cells.

### Analysis of SA β-galactosidase activity

**Figure 5.**
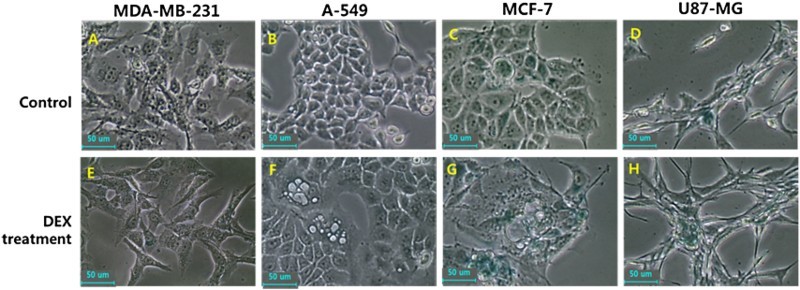
Activity of senescence-associated-β-galactosidase in untreated control (A, B, C and D) and 1 μg/ml DEX-treated (E, F, G and H) A-549, MDA-MB-231, MCF-7 and U87-MG cancer cells. Morphological change with lipid droplets were also observed, but change of senescence-associated-β-galactosidase activity not displayed by DEX treatment.

The activity of β-galactosidase for assessment of cellular senescence was investigated in DEX-treated MDA-MB-231, A-549, MCF-7 and U87-MG cancer cells and the results are shown in Figure 5. After DEX treatment for 2 weeks, the alternation of cells with adiposome-like vesicles were gradually displayed in A-549 and MCF-7as observed in Figure 3. However, the frequency of cells with high activity of SA β-gal was not observed in A-549, MDA-MB-231, MCF-7 and U87-MG cancer cells treated with 1 μg/ml DEX compared with those of untreated control cell lines implying that the cellular senescence might not be induced by DEX treatment.

### Analysis of telomerase activity

**Figure 6.**
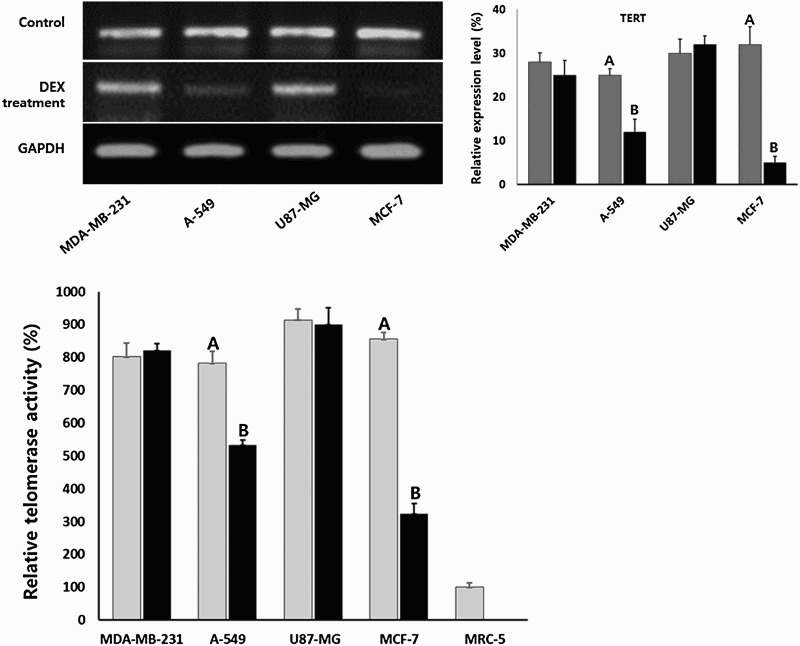
Expression level of TERT analyzed by RT-PCR (A) and telomerase activity (B) analyzed by RQ-TRAP (B) in untreated control (▪) and 1 μg/ml DEX-treated (▪) A-549, MDA-MB-231, MCF-7 and U87-MG cancer cells. A and B indicate significant (P<.05) difference between untreated control and DEX treatment, respectively.

The expression level of TERT transcript, tightly related with telomerase activity, was analyzed by RT–PCR and the telomerase activity was analyzed by real-time quantitative TRAP assay in MDA-MB-231, A-549, MCF-7 and U87-MG cancer cells treated with 1 μg/ml DEX, as shown in Figure 6. The expression level of TERT transcript (mean ± SEM) was 28 ± 2.1, 25 ± 1.5, 30 ± 3.2 and 32 ± 4.1% in the untreated control MDA-MB-231, A-549, MCF-7 and U87-MG cancer cells, respectively. However, the relative expression level of TRET transcript (mean ± SEM) was 25 ± 3.3, 6 ± 2.9, 32 ± 1.9 and 5 ± 1.5% in A-549, MDA-MB-231 and U87-MG cancer cells treated with DEX, respectively. The expression of TERT transcript by DEX treatment was significantly (*P* < .05) decreased in the A-549 and MCF-7 cancer cells with adiposome vesicles. Further, the expression level of telomerase activity in DEX-treated cancer cell lines was relatively compared with untreated normal MRC-5 fibroblasts (Figure 6). The relative level of telomere activity (mean ± SEM) was 801 ± 41.5, 782 ± 36.0, 913 ± 33.5 and 855 ± 19.3% in untreated control MDA-MB-231, A-549, MCF-7 and U87-MG, respectively. The high level of telomerase activity was observed in each cancer cell lines compared to normal MRC-5 fibroblasts. The relative level of telomerase activity was 821 ± 20.9, 532 ± 15.3, 900 ± 50.8 and 323 ± 30.9% in the A-549, MDA-MB-231 and U87-MG, MCF-7 treated with DEX, respectively. The expression of telomerase activity was also significantly (*P* < .05) decreased in A-549 and MCF-7 cancer cells with adiposome vesicles after DEX treatment.

## Discussion

In the present study, different types of human cancer cell lines (A-549, MDA-MB-231, MCF-7 and U87-MG), human normal MRC-5 fetal fibroblasts, umbilical cord matrix-derived mesenchymal stem cells (UCMSCs) and dental papilla tissue-derived mesenchymal stem cells (DSCs) were prolongedly exposed to 1 μg/ml of synthetic glucocorticoid dexamethasone (DEX) for up to 2 weeks. The cells were investigated for their cell proliferation, cellular differentiation into adipocytes, telomerase activity, and cellular senescence. The prolonged exposure to 1 μg/ml (∼0.25 μM) of DEX induced the inhibition of cell growth in cancer cell lines, such as A-549 and MCF-7 cancer cells. These results clearly demonstrated that the effect of DEX treatment on growth inhibition is accomplished by the cellular differentiation into adipocyte-like cells rather undergoing cell death by cell senescence and apoptosis.

In in vitro studies, the direct exposure to high concentration of GCs was resulted in delayed apoptosis and high survival rate by inhibition of caspase 3 activity in human and rat hepatocytes (Bailly-Maitre et al. 2001) and the inhibition of apoptosis by up-regulated caspase inhibitor, cIAP2, in ovarian cancer (Runnebaum and Brüning 2005). The GCs were also responsible for the induction of cellular apoptosis in osteoblasts and osteocytes subsequently resulting in decreased bone formation (osteoblastogenesis) and bone density in mice (Weinstein et al. 1998). Additionally, it has been reported that several types of normal and neoplastic lymphoid cells which are directly exposed to high concentrations of GCs displayed reduced cell growth due to the induction of programed cell death (Qian et al. 2011; Buxant et al. 2015; Jackson et al. 2016). In the present study, the inhibition of cell growth was also observed after DEX exposure in cancer cells, such as A-549 lung and MCF-7 breast adenocarcinoma. This is in accordance with the previous study in which DEX treatment was resulted in the anti-proliferation effect in MCF-7 breast cancer cell lines (Buxant et al. 2015). We have also observed that the extreme growth inhibition in DEX-treated mouse 3T3-L1 preadipocytes which was used as a control cells for adipgenesis. However, MDA-MB-231 cancer cells, MRC-5 normal fibroblasts, and mesenchymal stem cell lines including UCMSCs and DSCs have not shown the inhibition in cell growth. Whereas, the PDT analysis showed that U87-MG brain cancer cells treated with DEX undergone an increase of cell proliferation. The U87-MG brain cancer cells are a type of malignant glioma derived from neuroglia cells. Neurons in the brain depend upon glucose as their main source of energy, and one of the main functions of the neuroglia cells including astrocytes is the accumulation of glycogen for releasing glucose during periods of high rate of glucose consumption by cells leading to shortage of glucose (Bélanger et al. 2011). The cellular glucose flowed by various glucose transporters from blood is either used for energy production or mainly stored as triglycerides formed from glycerol and three fatty acids, and glycogen formed from multibranched polysaccharide of glucose. Although the glycogen accumulation in brain cells is very low when compared to liver, muscle, and other cells, glycogens are only stored in the brain cells predominately in astrocytes rather than triglycerides (Brown and Ransom 2007). In this study, the expression of GLUT4 transcript and ratio 0f glucose uptake were highly increased in U87-MG cancer cells after being exposed to DEX, while expression of PPARγ and GRβ transcripts was detected at very low level when compared to other cancer cell lines. The genes PPARγ and GRβ play an important role in the accumulation of triglycerides and adipogenesis (Rosen et al. 1999; Ali et al. 2013). Previous study has shown that the glycogen synthesis is induced by DEX treatment with noradrenaline in primary cultures of mouse cortical astrocytes (Allaman et al. 2004). Moreover, large amount of energy production is required for higher cell proliferation capacity. In the present study, high glucose uptake in most of the cancer cells was exhibited when compared to normal cell lines. Previously, it has been demonstrated that in H9c2 (2–1) cells, the cell proliferation was associated with an increased glucose uptake and energy metabolism through increased GLUT4 expression (Liu et al. 2013). However, the malfunction of GLUT4 was resulted in the inhibition of cell proliferation in U87-MG cancer cells (Azzalin et al. 2017). Thus, we assume that the highly accumulated cellular glucose by GLUT4 might be used for energy production required for cellular metabolism and growth rather relying on triglycerides converted through adipogenesis in in DEX-treated U87-MG cells.

The effect of growth inhibition by DEX was due to apoptosis in the earlier studies (Qian et al. 2011; Jackson et al. 2016). However, our results have demonstrated that growth inhibition is associated with the cellular differentiation into adipocyte-like cells rather than apoptosis in A-549 lung and MCF-7 cancer cells after being exposed to DEX at a concentration of 1 μg/ml. The high expression of SA β-gal activity is considered to be a marker of cellular aging and senescence preceding cellular apoptosis (Moon et al. 2015; Kim et al. 2017). In the present study, the activity of SA β-gal was not elevated in cancer cells treated with DEX, implying that the cellular senescence or apoptosis might not be induced by DEX exposure. However, we interestingly found that the growth inhibition is tightly associated with cellular differentiation into adipocyte-like cells. It has been known that the latent differentiation capacity into different cell types is also one of a tumorous characterization (Reya et al. 2001). In the current investigation, numerous small vesicles like adiposome are observed under microscope in the cytoplasm of DEX-treated A-549 and MCF-7 cancer cells showing retarded cell proliferation, and these vesicles were readily stained with Oil Red O solution, implying that the neutral triglycerides and lipids were accumulated in their cytoplasm and these cells were differentiated into adipocytes after DEX treatment. The DEX is an additive of differentiation cocktail generally used for adipogenic differentiation of stem cells along with insulin (Jeon et al. 2011b; Shivakumar et al. 2016). The mouse 3T3-L1 preadipocytes used as a control cells for adipogenesis in this study were also readily differentiated into adipocytes with massive accumulation of lipid molecules in the cytoplasm when cultured in the medium containing DEX and insulin, and similar observations were made in the previous studies (Chen et al. 2016). The A-DMEM is a widely applied basal medium that include insulin and other molecules for the in vitro cell culture of various mammalian cells. Our results have also shown that 3T3-L1 preadipocytes exposed to DEX are exceedingly induced to the adipocyte differentiation in the medium containing insulin. The analysis of glucose uptake and transcripts related to adipogenesis has further supported the cellular differentiation into adipocyte-like cells in DEX-treated A-549 and MCF-7 cancer cells. Previous studies have also demonstrated that the high glucose uptake is tightly associated with the up-regulation of insulin-dependent glucose transporter GLUT4 during the conversion of 3T3 fibroblasts into adipocytes (Lundgren et al. 2004). Our study has also shown that high glucose uptake is observed in the cancer cell lines with increased expression of GLUT4 than in normal cell lines. GLUT4 transmembrane protein induces the facilitated diffusion of circulating glucose into mainly adipose and muscle tissues by an insulin-activated manner (Huang and Czech 2007). Previous study has shown that GLUT4 transcripts were markedly increased after DEX treatment in combination with insulin rather than DEX or insulin-alone in 3T3 cells (Hajduch et al. 1995). In the present study, the remarkably increased glucose uptake is exhibited by all the cancer cell lines used, with elevated expression of GLUT4 and GRβ after DEX treatment. However, the cellular differentiation into adipocyte-like cells was readily induced in A-549 and MCF-7 cancer cells compared to MDA-MB-231 and U87-MG cancer cells, and the reason underlying these differences observed in this study is not clearly understood. The extra glucose which enters the cells is generally converted to glycogen or triglycerides in most of the cell types, except for brain tissue, as mentioned above. During adipogenesis, the cells developing into mature adipocytes is a well-regulated multistep process that requires the time-based activation of transcription factors. During adipogenesis, the first short wave induces several type of transcription factors including GR and others, and more importantly, the second long wave induces mainly PPARγ transcription factor, and therefore PPARγ is the most important modulator of adipogenesis (Ali et al. 2013; Lefterova et al. 2014). Glucocorticoids such as DEX, insulin and FBS are known to be adipogenic inducers by activating PPARγ transcription factor (Ali et al. 2013; Lefterova et al. 2014). Further, the activated PPARγ subsequently facilitates the accumulation of triglycerides (Medina-Gomez et al. 2007). In the present study, even though the glucose uptake, and the expression of GLUT4 and GRβ transcripts were highly elevated in cancer cells, the expression of PPARγ transcription factor was also extremely increased in A-549 and MCF-7 cancer cells. The differentiation into adipocyte-like cells and triglycerides accumulation upon DEX exposure may be thus due to the consequence of higher PPARγ expression in A-549 and MCF-7 cancer cells. And we assume that the inhibition of cell growth after DEX exposure is apparently associated with the loss of tumorous characteristics through cellular differentiation. The previous study has suggested that the inhibition of cell growth can results in the activation of PPARγ in several in vitro cultured human cancer cell lines (Krishnan et al. 2007).

Telomerase activity is essential for maintaining telomeric repeats and its high level activity in cancer cells results in acquisition of unlimited proliferation potential and cellular immortality without losing telomeric repeats (Maciejowski and de Lange 2017). The analysis of telomerase activity has shown to be upregulated in cancer cells compared to normal cell lines. However, the level of telomerase activity was gradually down-regulated in A-549 and MCF-7 cancer cells with reduced cell growth and adipocyte differentiation. The cellular differentiation of cancer cells generally associated with reduced telomerase activity (Sharma et al. 1995). Further, similar to cancer cells, the embryonic stem cells with high telomerase activity were also shown gradual loss of telomerase activity during the process of cellular differentiation (Odorico et al. 2001). Even though the exact mechanism underlying the effect of DEX treatment on telomerase activity in cancer cells is not known, we assume that the downregulation of telomerase activity is apparently associated with the loss of tumorous characteristics through cellular differentiation in A-549 and MCF-7 cancer cells after treating with DEX.

In conclusion, the present study has shown that stress-related corticosteroid hormone (glucocorticoid), DEX, evidently exhibits the inhibition of cell growth through induction of cellular differentiation into adipocyte-like cells in certain cancer cells such as A-549 and MCF-7 with highly increased glucose uptake and PPARγ expression. Therefore, it is important to find out a critical factors to predict DEX-responsiveness, and DEX could be an effective agent for treatment of certain cancers. However, exposure to DEX can cause certain side effects in the body such as suppression of immune system, decreased bone formation, and increased blood glucose. Thus, DEX treatment should be carefully investigated before conducting in vivo experiments or used in human subjects.
